# First Molecular Detection of *Echinococcus* *granulosus* Sensu Stricto in Dogs from Istanbul’s Anatolian Side: A Multi-methodological Approach

**DOI:** 10.1007/s11686-026-01283-4

**Published:** 2026-05-02

**Authors:** Mesut Akil, Mehmet Karakus, Sena Erkinay, Mehmet Aykur, Hakki Seckin Cetin, Muhammet Karakavuk, Tuncer Ozekinci, Esra Kocoglu

**Affiliations:** 1 Department of Parasitology, Faculty of Medicine, Istanbul Medeniyet University, 34700 Istanbul, Uskudar, Turkey; 2 Department of Medical Microbiology, Hamidiye Faculty of Medicine, University of Health Sciences, Istanbul, Turkey; 3 Department of Parasitology, Veterinary Faculty, Istanbul University-Cerrahpasa, Istanbul, Turkey; 4 Department of Parasitology, Faculty of Medicine, Tokat Gaziosmanpasa University, Tokat, Turkey; 5 ParsLab Veterinary Laboratory, Umraniye, Istanbul, Turkey; 6 Odemis Vocational School, Ege University, Izmir, Turkey; 7 Department of Medical Microbiology, Faculty of Medicine, Istanbul Medeniyet University, Istanbul, Turkey

**Keywords:** *Echinococcus granulosus*, Dogs, Copro-ELISA, Copro-PCR, Genotype, Istanbul

## Abstract

**Purpose:**

Cystic echinococcosis (CE) is a zoonotic disease caused by the larval stage of *Echinococcus granulosus* sensu lato (s.l.). Istanbul’s large stray dog population represents a significant risk for human CE transmission. However, prevalence and genotype studies about *E. granulosus* s.l. in dogs remain limited. This study aimed to determine the presence and genotypes of *E. granulosus* s.l. in stray dogs on the Anatolian side of Istanbul using microscopy, Copro-Enzyme-Linked Immunosorbent Assay (Copro-ELISA), and molecular diagnostic methods.

**Methods:**

Fecal samples (*n* = 110) were collected from stray dogs in seven districts. Samples were examined using modified formalin–ethyl acetate sedimentation (mFEAS). Coproantigens were detected using a commercial ELISA kit. Genomic DNA was extracted for PCR targeting the mitochondrial CO1 gene, and positive amplicons were sequenced and evaluated phylogenetically.

**Results:**

Microscopy revealed Taeniid-type eggs in 2 samples (1.8%) and these were confirmed molecularly. Copro-ELISA detected 7 positive samples (6.4%); however, none of these were confirmed by molecular and microscopy. Sequencing analysis revealed *E. granulosus* sensu stricto (s.s.) (G1 genotype) in 2 dogs from Sultanbeyli and Beykoz. Phylogenetic analysis demonstrated both isolates clustered within the *E. granulosus* s.s. (G1–G3) clade.

**Conclusions:**

This study provides the first molecular confirmation of *E. granulosus* s.s. in stray dogs on Istanbul’s Anatolian side, showing active zoonotic transmission potential in this highly populated region. The discrepancy between Copro-ELISA and molecular/microscopic findings highlights the requirement for multi-methodological approach. Continuing prevalence studies and molecular surveillance within a One Health framework are essential to reduce the public health threat posed by CE.

**Supplementary Information:**

The online version contains supplementary material available at 10.1007/s11686-026-01283-4.

## Introduction

Cystic echinococcosis (CE) is an important zoonotic disease caused by the larval stage of *Echinococcus granulosus* sensu lato (s.l.), which is a cryptic species complex [1]. This disease continues to pose serious health risks to both animals and humans; notably, recent epidemiological data highlight its persistent public health impact and distribution across Europe [2]. CE is more common in areas where the dog population and livestock farming are widespread. Therefore, it causes a considerable economic burden on the livestock industry and public healthcare sector [3]. According to its global impact, the World Health Organization listed CE as one of the most important food-borne parasitic diseases in 2014 [4].

Carnivores, particularly dogs, play a role as a definitive host in the domestic life cycle of *E. granulosus* s.l [5]. Additionally, a sylvatic cycle exists involving various wild canids such as dingoes and wolves serve as definitive hosts, while wild ungulates (moose, elk, red deer, and wild boar), and various marsupials, such as kangaroos, act as intermediate hosts. And this cycle varies by region, as observed in the Australian sylvatic cycle or the Northern form in North America and Eurasia [6, 7]. In both domestic and sylvatic life cycles, adult parasites are located in small intestine of definitive hosts, and the eggs of the parasite are spread to the environment via feces, contaminating soil, water, and food sources. Intermediate hosts, including livestock (sheep, cattle, and goat etc.), wild ungulates and humans can become infected by ingesting these eggs from contaminated sources [8]. Humans are accidental intermediate hosts. Once the infection is acquired from dogs, hydatid cysts develop primarily in the liver, secondarily in the lungs and then in other organs. Therefore, transmission risk is especially high in endemic areas with large stray dog populations, where the domestic cycle is actively maintained [3, 9].

Molecular studies have revealed that *E. granulosus* s.l. is a cryptic species complex including distinct genotypes and species with varying geographic distributions, host specificity, and pathogenicity. Within this complex, *E. granulosus* sensu stricto (s.s) (G1–G3) is recognized as the primary cause of cystic echinococcosis in human [10]. Phylogenetic studies have also identified other genotypes of the complex including *E. equinus* (G4), *E. ortleppi* (G5), *E. canadensis* (G6-G10) and *E. felidis* [5, 11].

In endemic regions, such as many parts of Türkiye, the large and growing stray dog population plays significant role in transmission of *E. granulosus* s.l. In molecular studies investigating genotypes of *E. granulosus* s.l. in Türkiye, the G1-G3, G4, G6, and G7 genotypes were identified in livestock animals, while the G1-G3, G4, G6, and G7 genotypes were detected in humans [12, 13]. The studies in dogs have also revealed the presence of G1-G3, G4, G5 and G6/G7 genotypes [14]. Despite these findings, data on the prevalence and genotype diversity of *E. granulosus* s.l. in dogs remains limited, particularly studies using advanced molecular approaches [8, 15].

Diagnosing *Echinococcus* infections in dogs is a crucial step for understanding the epidemiology and planning effective control programs. There are several diagnostic methods for the infection in dogs, each with advantages and disadvantages. Direct microscopy of dog feces is commonly used to investigate the presence of eggs or adult forms of the parasite. Although supplementary methods like flotation and sedimentation techniques can help in the detection of parasitic structures, enhancing the sensitivity of technique, it is limited by its inability to differentiate from Taeniid-type eggs [16, 17]. The detection of *E. granulosus* s.l. antigens in feces using Copro-Enzyme-Linked Immunosorbent Assay (Copro-ELISA) provides a more reliable method than serological tests in dogs [18]. This immunological approach enhances diagnostic specificity and sensitivity, particularly in field studies or regions with endemic infections. Furthermore, molecular techniques, including polymerase chain reaction (PCR) and DNA sequencing, have significant role in identifying and characterizing *E. granulosus* s.l. genotypes. These advanced tools not only facilitate precise diagnosis but also contribute to epidemiological studies by revealing the genetic diversity and geographical distribution of the parasite [19]. In this case, using a combination of microscopy, Copro-ELISA, and PCR provides a complementary diagnostic framework for CE surveillance in dogs [20].

To date, studies investigating the prevalence of *Echinococcus* species in Istanbul remain limited. In a coprological survey of stray dogs and cats, it was reported that *E. granulosus* s.l. in 0.8% of dogs, based on only microscopic and macroscopic identification [21]. In addition, a molecular study reported from Istanbul with human surgical isolate which was identified as *E. granulosus* s.s., genotype G1 [22]. However, no molecular studies have been conducted on dogs to confirm species and genotype distribution, despite their key role in environmental contamination and transmission. Considering Istanbul’s large stray dog population, detection of the prevalence and genotypes *E. granulosus* s.l. in dogs is essential for understanding transmission dynamics.

Therefore, in this study we aimed to determine the prevalence of *E. granulosus* s.s. in stray dogs from selected districts of Istanbul’s Anatolian Side using a combination of direct microscopy, Copro-ELISA, and molecular analysis.

## Material and Method

### Study Area

**Fig. 1:**
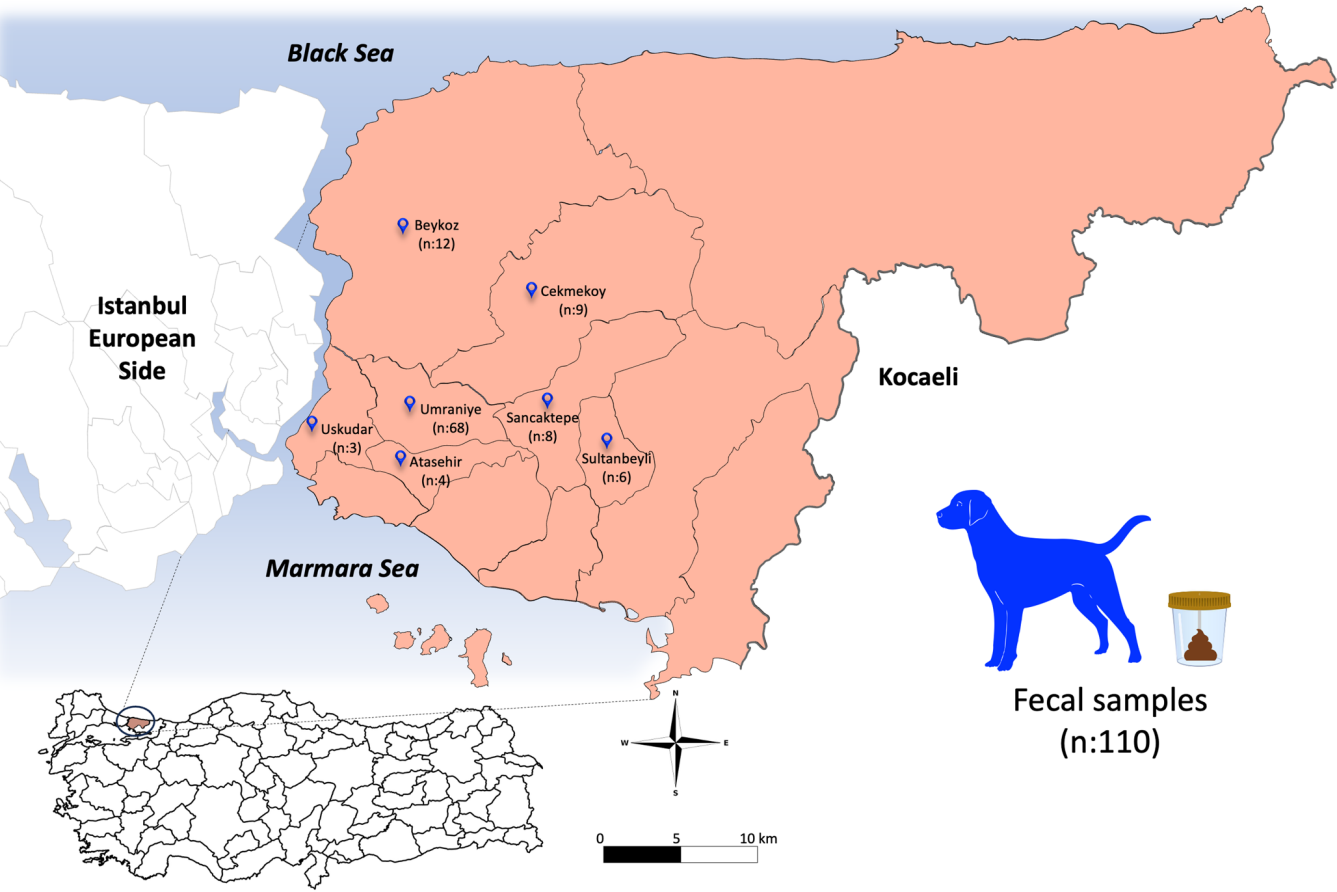
Distribution of collected samples from the dogs in the Anatolian Side of Istanbul

The study was conducted in Istanbul Province (40°58′53″N, 29°05′41″E), located at the junction of the Asian and European continents, is the most populous city in Türkiye. In addition, according to 2018 estimates, approximately 130,000 stray dogs inhabit the city [23]. The geographic origins of the dogs are shown on the map according to their locations which including 7 districts in the sampling: Umraniye (*n* = 68), Beykoz (*n* = 12), Cekmekoy (*n* = 9), Sancaktepe (*n* = 8), Sultanbeyli (*n* = 6), Atasehir (*n* = 4), and Uskudar (*n* = 3) (Fig. 1).

#### Sample Collection

A total of 110 fecal samples were collected between April 2022 and November 2022 from dogs brought to municipal shelters or private veterinary clinics for routine anti-parasitic treatment and sterilization procedures. Due to field conditions and accessibility limitations, a convenience-based opportunistic sampling approach was utilized, which accounts for the unbalanced number of samples across the study areas [6, 24]. All dogs included in the study were over one year of age to better assess the public health risk and potentially infected animals. And their estimated ages were recorded based on dental examination.

Following anthelmintic treatment, fecal samples were collected after the first defecation within 24 h. Each sample gathered in a sterile, leak-proof containers which was labelled with unique identification number and was brought to the laboratory under cold chain conditions. Then, samples initially stored at − 80 °C for minimum 7 days to inactivate potential infective parasite eggs. After the inactivation period, samples were kept at − 20 °C until further examinations and analysis [25].

##### Microscopic Examination

All samples were examined for the presence of Taeniid-type eggs using the modified formalin–ethyl acetate sedimentation (mFEAS) techniques in order to increase diagnostic sensitivity. This concentration technique was performed according to standard parasitological protocols to enhance detection sensitivity [26–29]. The main steps of the procedure are illustrated in Supplementary Fig. 1. The procedure involves fixation in 10% formalin, filtration, addition of ethyl acetate, centrifugation, and microscopic examination of the sediment using physiological saline.

#### Copro-ELISA

To detect *Echinococcus*-specific coproantigens, the Canine *Echinococcus* Antigen ELISA kit (Elabscience, China) was used according to the manufacturer’s instructions. Briefly, approximately 1 g of fecal sample was diluted in 1 mL of sample solution provided within the kit and mixed vigorously for 5 min. The suspension was then centrifuged at 3000 rpm for 10 min, and the supernatant was collected.

Aliquots of 100 µL of each sample, along with positive and negative controls, were added in duplicate to the ELISA plate wells. After incubation at 37 °C for 30 min in the dark, plates were washed five times at 30-second intervals using the provided washing buffer. Subsequently, 100 µL of HRP enzyme conjugate was added to each well, followed by another 30 min incubation at 37 °C in the dark. The washing step was then repeated. After that, 100 µL of substrate reagent was added and incubated for 10 min at 37 °C. Then, reaction was finished with stop solution.

The optical density (OD) values were measured at 450 nm using a microplate spectrophotometer. According to the manufacturer’s criteria, samples with OD values exceeding the calculated cut-off (mean OD of negative controls + 2.1 × SD) were considered positive for *Echinococcus* antigens.

### Molecular Analysis

Total genomic DNA was extracted from each fecal sample using the Quick-DNA™ Fecal/Soil Microbe Miniprep Kit (Zymo Research, USA) according to the manufacturer’s protocol. Before extraction, in order to disrupt the possible eggshells and release DNA in parasite eggs, ZR BashingBead Lysis system in a Qiagen TissueLyser LT was performed at high frequency for 10 min 3 times. The extracted DNA was stored at -20 °C until PCR amplification.

PCR reaction was performed with 12.5 µL Xpert Fast HotStart Master Mix (2x) (GRISP, Portugal) 2 µL of each primer (10 pmol), 3.5 µl nuclease-free water and 5 µl template DNA. Primer pair is JB3 (5′-TTTTTTGGGCATCCTGAGGTTTAT-3′) and JB4.5 (5′-TAAAGAAAGAACATAATGAAAATG-3′), which amplify about 450 bp fragment of the mitochondrial cytochrome c oxidase subunit 1 (CO1) gene [30].

PCR amplification was performed in a thermal cycler using the following profile: initial denaturation at 95 °C for 5 min, followed by 40 cycles of denaturation at 95 °C for 15 s, annealing at 50 °C for 15 s, and extension at 72 °C for 5 s, with a final extension at 72 °C for 5 min. A previously confirmed *E. granulosus* s.s. (Genotype 1) DNA sample in our laboratory was used as a positive control, and nuclease-free water was included as a negative control.

For DNA visualization, Xpert Green DNA Stain direct (GRiSP, Portugal) was used as a safe, non-mutagenic alternative to ethidium bromide. PCR products were mixed with the stain in a 10:1 ratio (10 µL of sample to 1 µL of stain) and loaded directly onto the gel without additional loading buffers. PCR products were electrophoresed on 1.5% agarose gels in 1× TAE buffer at 120 V for 1 h and visualized under UV illumination. Amplicons displaying about 450 bp band were purified and submitted to commercial firm (Medsantek, Istanbul, Türkiye) for bidirectional Sanger sequencing. Raw chromatograms were edited and assembled. Consensus sequences were compared with reference sequences in GenBank using the BLASTn algorithm to confirm species identity. A phylogenetic tree was constructed using the Neighbor-Joining method under the Kimura 2-parameter model with a gamma distribution in MEGA X software, incorporating *E. granulosus* s.l. genotype sequences obtained in this study along with reference sequences retrieved from the GenBank database [31]. The reliability of the phylogenetic tree was evaluated using 1000 bootstrap replicates. A sequence of *Taenia multiceps* (GenBank accession number: AB792725) was employed as an outgroup.

### Ethics Approval

Ethical approval for this study was obtained from the Istanbul-Cerrahpaşa Local Ethical Committee for Animal Experiments (decision number E-74555795-050.01.04-379648), which states that formal ethical clearance is not necessary for the collection of fecal samples from dogs according to institutional and national regulations due to fecal collection was required non-invasive manner without causing distress to the animals.

### Statistical Analysis

All data were entered and organized using Microsoft Excel. Descriptive statistics were calculated, with categorical variables presented as frequencies and percentages, and continuous variables as mean ± standard deviation. The 95% confidence intervals (CIs) for the prevalence rates were calculated using the exact binomial (Clopper-Pearson) method. Associations between *Echinococcus* positivity and groups were analyzed using the chi-square test and a Z-test was applied to compare the infection percentages between subgroups. Agreement between diagnostic methods (microscopy, Copro-ELISA, and PCR) was evaluated using Cohen’s kappa. A significance level of *p* < 0.05 was considered statistically significant. All statistical analyses were performed using IBM SPSS Statistics version 22.

## Results

Of the 110 dogs sampled, 60 were male (54.5%) and 50 were female (45.5%) (Table 1). The estimated mean age was 3.34 ± 2.51 years (range 1 to ≥ 5). Age distribution was: 1 year, 21 (19.1%); 2 years, 40 (36.4%); 3 years, 6 (5.5%); 4 years, 9 (8.2%); ≥5 years, 34 (30.9%). There was no statistically significant association between *Echinococcus* positivity and gender or age group (*p* > 0.05).

**Table 1:** Gender and estimated age distribution of the dogs

Variable	*N*	Proportion(%)
*Gender*		
Male	60	54.5%
Female	50	45.5%
*Age (years)*		
1	21	19.1%
2	40	36.4%
3	6	5.5%
4	9	8.2%
≥ 5	34	30.9%

### Microscopic Examination

Of the 110 fecal samples examined using microscopy, Taeniid-type eggs were detected in 2 samples (1.8%; 95% CI: 0.2%–6.4%) using mFEAS methods. Due to the morphological similarity of Taeniid-type eggs, species level identification was not possible with microscopy alone. Taeniid-type eggs and artefacts are shown in Supplementary Fig. 2. Microscopy-positive samples were confirmed as *E. granulosus* s.s. by molecularly and there was a 100% agreement between microscopy and PCR findings.

### Copro-ELISA

Copro-ELISA detected *Echinococcus*-specific antigens in 7 of the 110 samples, corresponding to a prevalence of 6.4% (95% CI: 2.6–12.8%). In addition, there was no statistically significant difference in the Copro-ELISA positivity between females (4/50, 8.0%) and males (3/60, 5.0%) (two-proportion Z-test, Z = 0.64, *p* = 0.52). Notably, the two samples that were positive for Taeniid-type eggs by microscopy tested negative in Copro-ELISA.

The geographical distribution of ELISA positive samples was shown in Table 2. According to ELISA results, positive samples were observed in Umraniye (*n* = 3), Sancaktepe (*n* = 1), Sultanbeyli (*n* = 2) and Uskudar (*n* = 1). No significant association was found between ELISA positivity and location (*p* > 0.05).

**Table 2:** Distribution of Copro-ELISA positive samples by district on the Anatolian side of Istanbul

District	No. samples	ELISA positive (*n*)	Prevalence% (95%CI)
Ümraniye	68	3	4.4 (0.9–12.4%)
Beykoz	12	0	0 (0.0–26.5%)
Çekmeköy	9	0	0 (0.0–33.6%)
Sancaktepe	8	1	12.5 (0.3–52.7%)
Sultanbeyli	6	2	33.3 (4.3–77.7%)
Ataşehir	4	0	0 (0.0–60.2%)
Üsküdar	3	1	33.3 (0.8–90.6%)
Total	110	7	6.4 (2.6–12.8%)

### Molecular Analysis

**Fig. 2:**
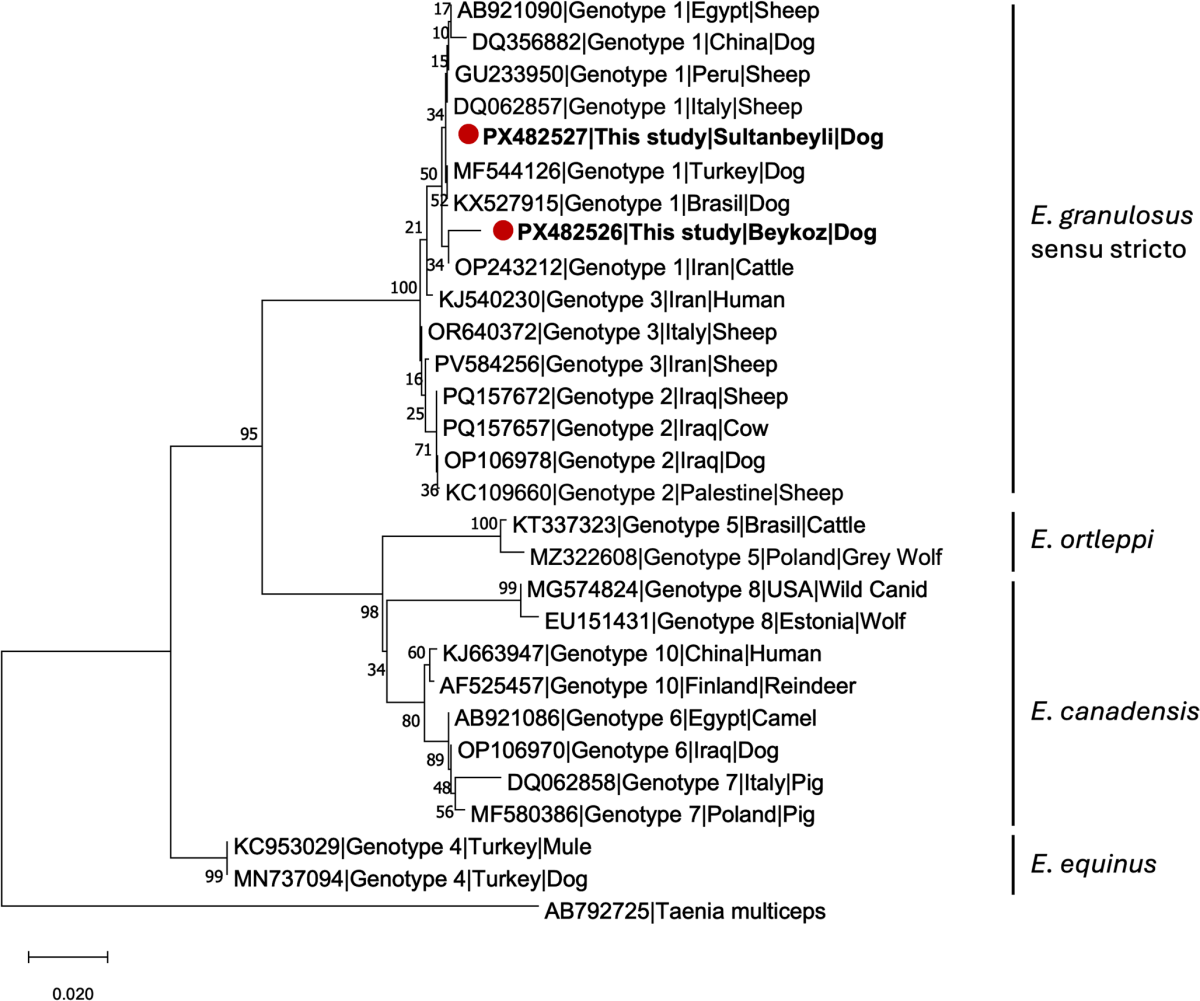
Phylogenetic tree of the reference sequences constructed using MEGA X software with the Neighbor-Joining method. Bootstrap analysis was performed with 1,000 replicates. Red circles indicate the sequences obtained in the present study

PCR amplification targeting about 450 bp fragment of the mitochondrial CO1 gene was performed on all 110 fecal samples using JB3/JB4.5 primers. Following the Sanger sequencing (Medsantek, Istanbul, Türkiye) of amplicons displaying a band of approximately 450 bp, no other taeniid DNA was detected. Sequence analysis revealed that only two samples, from Sultanbeyli and Beykoz districts, showed > 99% identity with reference *E. granulosus* s.s. (G1 genotype) sequence in GenBank (e.g. MF544126, MN732801). These sequences were deposited in GenBank with the accession numbers PX482526 (Beykoz) and PX482527 (Sultanbeyli). Phylogenetic analysis of the obtained sequences revealed genetic clustering within the *E. granulosus* s.l. complex, confirming that both dog isolates from this study belong to *E. granulosus* s.s. (G1–G3) (Fig. 2). The molecular findings supported microscopic detection of taeniid-type eggs, confirming the presence of *E. granulosus* s.s. in 2 of 110 (1.8%) in Anatolian side of Istanbul.

### Comparison of Diagnostic Methods

Since PCR and microscopy results are 100% consistent with each other, the results of both tests were accepted as the reference standard. The diagnostic performance of the Copro-ELISA was evaluated according to these tests results. Because the two microscopy and PCR-confirmed positive samples tested negative by Copro-ELISA, and the seven ELISA-positive samples could not be molecularly confirmed, the Copro-ELISA demonstrated a sensitivity of 0% and a specificity of 93.5% (95% CI: 87.0–97.3%). The positive predictive value was 0%, and the negative predictive value was 98.1% (95% CI: 93.2–99.8%). Consequently, Cohen’s Kappa analysis indicated no agreement between the Copro-ELISA and microscopy/PCR methods in this study (ϰ < 0).

## Discussion

*E. granulosus* s.l., the causative agent of CE, remains one of the most common zoonotic infections in both worldwide and Türkiye, where dogs act as definitive hosts and continually contaminate the environment by shedding parasitic eggs. Stray dogs play a critical role in maintaining transmission cycles causing infection in livestock and humans. Understanding the prevalence and genotypes circulating in canine populations is therefore essential for effective CE surveillance and control [7, 32]. Although various studies from Türkiye have investigated canine echinococcosis using various diagnostic tools with prevalences ranging from 0% to 29.3% depending on the region [14, 15, 28, 33–36], the data from Istanbul remain very limited. Oter et al. reported *E. granulosus* infection in 0.8% of stray dogs using only coprological examination (macroscopy of adult worms); however, no molecular confirmation or genotyping was performed [21]. The present study therefore provides the first molecular evidence confirming the presence of *E. granulosus* s.s. (G1 genotype) in stray dogs from Istanbul’s Anatolian side.

It is reported that there are over one million people infected with CE worldwide [37], and several rural regions of Türkiye remain highly endemic for the disease. According to an epidemiological study, the annual incidence of human CE has been estimated to range between approximately 0.8 and 6.4 per 100,000 people [32]. In Istanbul, in a forensic autopsy study conducted between 2003 and 2004, cyts was macroscopically detected in 56 of 1687 autopsies (3.3%) and 60.7% of these cases were confirmed as CE by serological tests [38]. In addition, 30 CE patients were identified in a single tertiary hospital in Istanbul between 2018 and 2023 [39]. These reports show that CE is still present in Istanbul and that ongoing transmission—most likely from infected dogs—continues to affect human cases.

In this study, *E. granulosus* s.s. DNA was detected in 1.8% of the dogs using both molecular and microscopic methods. This low rate is consistent with findings from other metropolitan areas where dogs have limited access to infected livestock offal. Similar findings have been reported from urban or semi-urban regions such as Mazandaran, Northern Iran (1.09%) [40]; Acre, Brazil (1.53%) [41]; Tartu, Estonia (2.2%) [42]; Tirana, Albania (2.7%) [43]; Kerman, South-eastern Iran (6.8%) [44]. The low prevalence observed in our study may be associated with factors such as urbanization, the management of stray dogs, and routine antiparasitic administrations in municipal shelters. Notably, the positive dogs were from Sultanbeyli and Beykoz, semi-rural districts where votive religious slaughtering is common. In these areas, stray dogs may still access cystic organs after slaughtering.

The multi-methodological approach used in this study highlights important differences among diagnostic tools. While microscopy and molecular methods detected *E. granulosus* s.s. in only 2 of samples (1.8%), Copro-ELISA detected *Echinococcus*-specific antigens in 7 of samples (6.4%), yielding a higher positivity rate than the other two methods. However, although the higher number of reactive samples were yielded by ELISA, two samples confirmed by both microscopic and molecular methods were negative. This indicates that these additional reactive results may represent false positives, questioning the assay’s perceived superior sensitivity in comparison to molecular tools.

Similar articles where ELISA detected higher prevalence rate than Copro-DNA detection have been reported in Kazakhstan [45], Tunisia [18] and Romania [46]. This discrepancy is often attributed to antigen persistence, early infections, or cross-reactivity with other cestodes such as *Taenia hydatigena* and *Dipylidium caninum* [47] However, the absence of other tapeworm eggs in the microscopic examination of our samples reduces the possibility of the cross reactivity hypothesis. This suggests that other, unknown cross reacting antigens may be responsible for the potential false positive ELISA outcomes. Furthermore, the lack of Copro-ELISA positivity in the two confirmed samples may be attributed to antigen degradation due to freeze-thawing or a low worm burden [48–50].

On the other hand, the failure to molecularly confirm the ELISA-positive samples could be strongly related to PCR inhibition [51]. Fecal samples represent a highly complex matrix, and although a specialized stool DNA extraction kit (Quick-DNA™ Fecal/Soil Microbe Miniprep Kit, Zymo Research, USA) was utilized to reduce the negative effects of inhibitors in this study, such kits are rarely 100% effective [52]. Furthermore, since an internal PCR amplification control was not used in present study to observe possible inhibition, this raises concerns about the possibility of false negative PCR results. To address this limitation, it is recommended that future molecular analyses of stool samples include internal amplification controls to monitor for potential non-specific inhibitors. Consequently, these factors likely contribute to the observed inconsistency between these assays. This conclusion is supported by diagnostic comparison, which showed no statistical agreement and low sensitivity for the Copro-ELISA in this sample set. Together, these findings highlight the limitations of using a single diagnostic method and emphasize the necessity to use of utilizing combined testing approaches in epidemiological surveys.

Sequence analysis confirmed that the detected isolates belonged to *E. granulosus* s.s. (G1 genotype). This genotype has been reported the main cause of human and livestock CE in Türkiye and worldwide [10, 12, 53]. The detection of G1 genotype in dogs from Istanbul indicates suggests that the sheep- dog transmission cycle remains active, even in a large urban environment. Notably, the same genotype was previously identified in human surgical samples in Istanbul [22]. This indicates that human infections in the region originate from local dogs, highlighting zoonotic risk of stray dogs. However, there is still no extensive investigation on livestock animals in Istanbul and further studies is required to better understand transmission in the region. Determination of the dominant in the endemic areas genotypes is essential for providing effective control programmes in terms of one health concept [35, 54].

## Conclusions

This study provides the first molecular identification of *E. granulosus* s.s. in stray dogs in Istanbul, addressing a gap in the epidemiology of CE in this region. The detection of this zoonotic genotype highlights the continued risk of transmission in urban and peri-urban districts. Considering the multi-methodological approach evaluated, more reliable findings were detected by molecular and microscopic methods compared to ELISA. Nevertheless, using microscopy, Copro-ELISA, and molecular methods together remains valuable for comprehensive diagnosis and should be applied in future surveillance programs. Improving routine antiparasitic treatment, stray dog management, and wasting management in livestock offal is important for reducing environmental contamination and support One Health-based control strategies.

## Supplementary Information

Below is the link to the electronic supplementary material.

Supplementary Material 1: Supplementary Fig. 1 Steps of the modified formalin–ethyl acetate sedimentation(mFEAS) technique used for the detection of Taeniid-type eggs in fecal samples

Supplementary Material 2: Supplementary Fig. 2 Microscopic observations (×400) of Taeniid-type eggs (A–B) andartefacts (C–E)

## Data Availability

The DNA sequence data generated in this study have been deposited in the GenBank database under accession numbers PX482526 and PX482527. All other reference sequences are included in the article.
