# Supplementary figures and images for: First Molecular Detection of Echinococcus granulosus Sensu Stricto in Dogs from Istanbul’s Anatolian Side: A Multi-methodological Approach
Source: Acta Parasitol. 2026 May 2;71(3):94. doi: 10.1007/s11686-026-01283-4 (PMC13135559; doi:10.1007/s11686-026-01283-4)

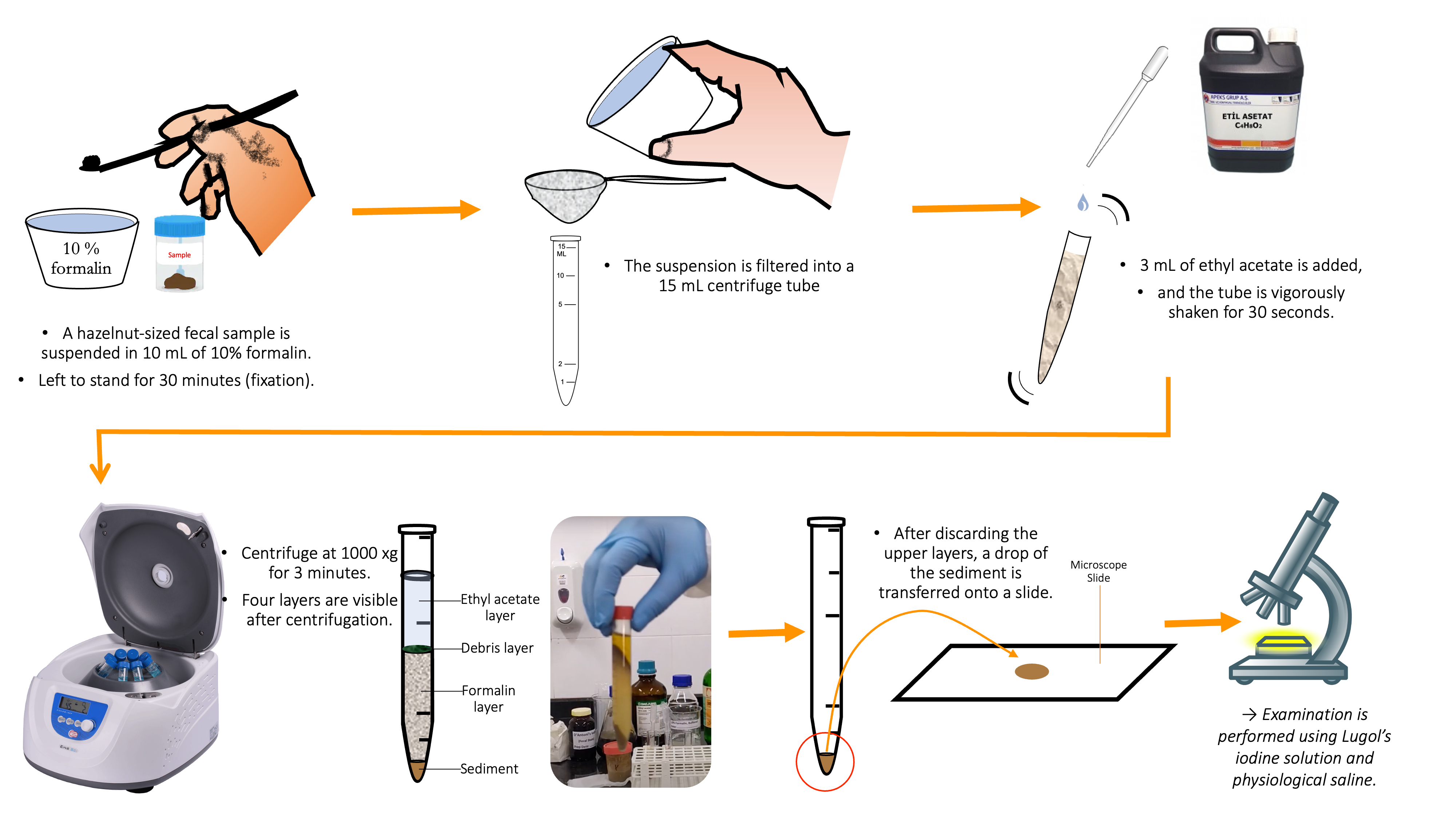

Supplement: Supplementary file 1 — Supplementary Material 1: Supplementary Fig. 1 Steps of the modified formalin–ethyl acetate sedimentation(mFEAS) technique used for the detection of Taeniid-type eggs in fecal samples [file 11686_2026_1283_MOESM1_ESM.tiff]

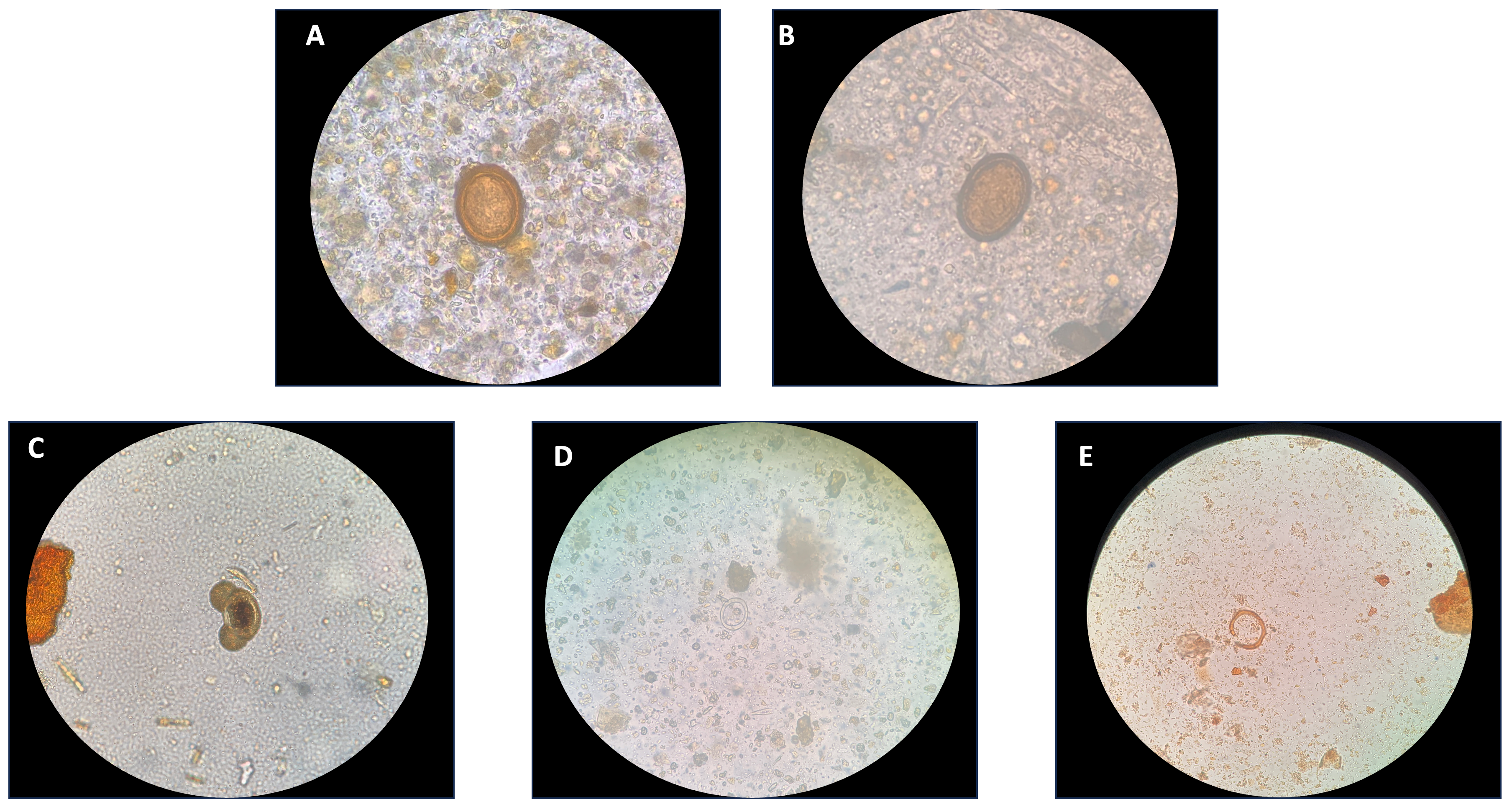

Supplement: Supplementary file 2 — Supplementary Material 2: Supplementary Fig. 2 Microscopic observations (×400) of Taeniid-type eggs (A–B) andartefacts (C–E) [file 11686_2026_1283_MOESM2_ESM.tiff]
